# Insulin-Like Growth Factor (IGF)-I and Insulin in Normal and Growth-Restricted Mother/Infant Pairs

**DOI:** 10.1155/2007/42646

**Published:** 2007-03-13

**Authors:** Ariadne Malamitsi-Puchner, Despina D. Briana, Dimitrios Gourgiotis, Maria Boutsikou, Karl-Philipp Puchner, Stavroula Baka, Antonios Marmarinos, Dimitrios Hassiakos

**Affiliations:** ^1^Neonatal Division, Second Department of Obstetrics and Gynecology, Athens University Medical School, 10682 Athens, Greece; ^2^Research Laboratories, Second Department of Pediatrics, Athens University Medical School, 11527 Athens, Greece

## Abstract

Insulin-like growth factor (IGF)-I and insulin are essential for fetal growth. We investigated perinatal changes of both factors in 40 mothers and their 20 appropriate-for-gestational-age (AGA) and 20 intrauterine-growth-restricted (IUGR) fetuses and neonates on day 1 (N1) and day 4 (N4) postpartum. Fetal and N1, but not N4, IGF-I levels were increased in AGA (*P* < .001 and *P* = .037, resp.). N1 insulin levels were lower in IUGR (*P* = .048). Maternal, fetal, and N1 IGF-I, and fetal insulin levels positively correlated with customized centiles (*r* = .374, *P* = .035, *r* = .608, *P* < .001, *r* = .485, *P* = .006, and *r* = .654, *P* = .021, resp.). Female infants presented elevated fetal and N4 IGF-I levels (*P* = .023 and *P* = .016, resp.). Positive correlations of maternal, fetal, and neonatal IGF-I levels, and fetal insulin levels with customized centiles underline implication of both hormones in fetal growth. IUGR infants present gradually increasing IGF-I levels. Higher IGF-I levels are documented in females.

## 1. INTRODUCTION

Fetal and neonatal growth is a complex process involving genetic, nutritional, hormonal, and environmental factors [1]. It is out of doubt that intrauterine growth restriction (IUGR) is associated with increased morbidity not only in the neonatal period, but also in childhood and later in adult life [1–3]. However, despite extensive research, the regulation of human fetal growth still remains unclear [1].

Two peptide hormones, that share structural homology, (insulin-like growth factor (IGF)-I and insulin), seem to be the most important endocrine regulators of fetal growth [4]. IGF-I, which is produced by fibroblasts and other cells of mesenchymal origin, has many mitogenic actions including stimulation of cellular growth, proliferation, and differentiation [5]. It also shows anabolic effects, enhancing glucose and amino acid uptake [6] and appears to be the dominant growth-promoting factor during the rapid phase of somatic growth in late gestation [7]. In the fetus, IGF-I concentrations are influenced by nutrients delivered through the placenta rather than by fetal pituitary growth hormone (GH) [8].

The role of insulin in fetal growth has also been clearly identified [9]. Insulin may augment fetal growth by stimulating the production of IGF-I [8]. Fetal insulin and IGF-I act synergistically to enhance uptake and utilization of substrates by the fetal tissues [8]. In IUGR pregnancies, changes of the insulin substrate and IGF-I can be largely attributed to poor nutrition with the majority of studies demonstrating low serum IGF-I and insulin levels in late gestation and at birth [10].

In postnatal life, nutrition, insulin, and IGF-I still largely regulate growth [11]. Circulating IGF-I levels increase rapidly after birth, primarily as a result of the onset of GH-stimulated IGF-I production by the liver [8]. This transient GH hypersecretion may drive the early growth acceleration that occurs to some degree in virtually all SGA infants [10].

This study explored, for the first time to our knowledge, the concomitant changes of these two peptide hormones in mother/infant pairs from IUGR and appropriate-for-gestational-age (AGA) pregnancies, at crucial time points of the perinatal period, characterizing intrauterine life, as well as transition and stabilization to extrauterine one.

## 2. SUBJECTS AND METHODS

The Ethics Committee of our teaching hospital approved the study protocol. Signed informed consent was acquired from all participating mothers. Forty parturients giving consecutively birth either to 20 AGA or 20 asymmetric IUGR full-term singleton infants with a birth weight below or equal to the 3rd customized centile were included in the study. The gestation-related optimal weight (GROW) computer-generated program [12, 13] was used to calculate the customized centile for each pregnancy, taking into consideration significant determinants of birth weight, as maternal height and booking weight, ethnic group, parity, gestational age, and gender [12]. Gestational age was estimated using the date of the last menstrual period and early antenatal ultrasound. Birth weight was measured with an electronic scale.

Nine of the 20 mothers with IUGR offspring presented preeclampsia [14]. The remaining 11 mothers presented pregnancy-induced hypertension and suffered from various pathological conditions, such as iron-deficient anemia (3 cases), gestational diabetes mellitus (2 cases), hypothyroidism (3 cases), extreme obesity (2 cases), and cardiac arrhythmias (1 case). Five of the above women were smoking more than 10 cigarettes per day during the whole duration of pregnancy.

Doppler studies were performed in the IUGR group every 10–15 days, starting from the 32nd gestational week. Doppler studies of the uterine and umbilical arteries were serially found to be in the upper physiological limits for gestational age in 13 cases, while in the remaining seven cases they showed increased impedance to flow (pulsatility index values greater than the 95th percentile for the corresponding gestational age). Regarding middle cerebral arteries, resistance was in the lower physiological limits for gestational age indicating the initiation of blood flow redistribution process. Nevertheless, amniotic fluid was diminished in all IUGR cases. For the evaluation of the amniotic fluid, the largest fluid column on the vertical plane was assessed and was defined as diminished, if <2 cm. Furthermore, placental weight was reduced ranging from 255 to 400 g.

In contrast, in the AGA group, mothers were healthy and were either nonsmokers or abstained from smoking during pregnancy. Moreover, placentas were normal in appearance and weight.

All neonates of both groups had no history of abnormal perinatal clinical course, (e.g., perinatal asphyxia and infection) or identifiable congenital malformation and inherited metabolic or genetic disorders. One- and five-minute Apgar scores were ≥ 8 in all IUGR cases and AGA controls. All neonates were breastfed and they all adapted well to extrauterine life with no signs of respiratory distress, lethargy, irritability, or poor feeding. The demographic data of participating infants and their mothers are listed in Table 1.

Blood was collected in pyrogen-free tubes from (i) mothers during the first stage of labor, or before receiving anesthesia in cases of elective cesarean section; (ii) the doubly clamped umbilical cords, reflecting fetal state (mixed arteriovenous blood); and (iii) neonates on postpartum day 1 (N1) and day 4 (N4), characterizing transition and stabilization to extrauterine life, respectively. Blood was allowed to clot and was immediately separated by centrifugation. The supernatant serum was kept frozen at −80°C until assay.

IGF-I was measured by ELISA (Assay Designs Inc., 800 Technology Drive Ann Arbor, Mich, USA). The minimum detectable concentration, intra- and interassay coefficients of variation (CV)% were 187 pg/mL, 4.9% and 7.1%, respectively.

The determination of insulin levels was performed by Microparticle Enzyme Immunoassay (Abbot Diagnostics (Axsym System), Wiesbaden, Germany). The minimum detectable concentration, intra- and interassay CV% were <1 *μ*U/ml, 4.1% and 5.3%, respectively.

## 3. STATISTICAL ANALYSIS

IGF-I data presented normal distribution (Kolmogorov-Smirnov test). The repeated measures analysis of variance with Bonferroni correction for multiple comparisons was used to assess statistically significant differences between the four measurements (maternal, fetal, N1, N4) of IGF-I levels. Insulin levels were not normally distributed (Kolmogorov-Smirnov test); therefore, nonparametric procedures (Mann-Whitney U test and Wilcoxon sum-rank test) were used. Pearson or Spearman's correlation coefficient, where appropriate, was used to detect positive or negative correlations. Values of *P* < .05 were considered statistically significant.

## 4. RESULTS

**Figure 1:**
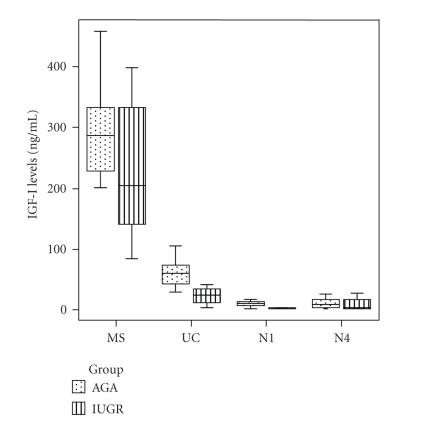
Box and whiskers plots of the concentration of IGFI in maternal (MS), fetal (UC), neonatal day-1 (N1), and day-4 (N4) serum samples from appropriate-for-gestational-age (AGA) and intrauterine-growth-restricted (IUGR) groups. Each box represents the median concentration with the interquartile range (25th and 75th percentiles). The upper and lower whiskers represent the range.

In the AGA group, maternal IGF-I levels were elevated compared to fetal (*P* < .001) and neonatal day-1 (*P* < .001) and day-4 (*P* < .001) levels after adjustment for multiple comparisons (Figure 1). Similarly, fetal IGF-I levels were significantly elevated compared to neonatal day-1 (*P* < .001) and day-4 (*P* < .001) (Figure 1).

In the IUGR group, maternal IGF-I levels were significantly elevated compared to fetal (*P* < .001), neonatal day-1 (*P* < .001) and day-4 (*P* < .001) levels after adjustment for multiple comparisons (Figure 1). Fetal IGF-I levels were significantly elevated compared to neonatal day-1 (*P* < .001) and day-4 (*P* = .002) IGF-I levels (Figure 1). Additionally, maternal insulin levels were significantly elevated compared to fetal (*P* < .001), neonatal day-1 (*P* = .001) and neonatal day-4 (*P* = .022) levels.

**Figure 2:**
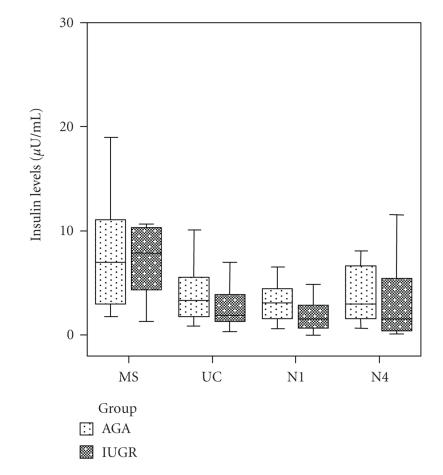
Box and whiskers plots of the concentration of insulin in maternal (MS), fetal (UC), neonatal day-1 (N1), and day-4 (N4) serum samples from appropriate-for-gestational-age (AGA) and intrauterine-growth-restricted (IUGR) groups. Each box represents the median concentration with the interquartile range (25th and 75th percentiles). The upper and lower whiskers represent the range.

Concerning differences between the groups, fetal and neonatal day-1 IGF-I levels were significantly increased in the AGA group (by 32.6 ng/mL and 4.2 ng/mL on average, resp.) compared to the IUGR one, after controlling for gender and adjusting for multiple comparisons (regression coefficient *b*: 32.6, SE: 7.4, *P* < .001, 95% confidence interval (CI): 17.5–47.7, and regression coefficient *b*: 4.2, SE: 1.9, *P* = .037, 95% CI: 0.3–8.2, resp.). No significant differences were found in neonatal day-4 IGF-I levels between the two groups. Neonatal day-1 insulin levels were significantly lower in IUGR neonates compared to AGA ones (*P* = .048) (Figure 2).

Maternal, fetal, and neonatal day-1 IGF-I levels positively correlated with customized centiles (*r* = .374, *P* = .035, *r* = .608, *P* < .001, and *r* = .485, *P* = .006, resp.). Moreover, fetal insulin levels positively correlated with customized centiles (*r* = .654, *P* = .021). In the AGA group, maternal insulin levels positively correlated with maternal IGF-I levels (*r* = .606, *P* = .037).

**Figure 3:**
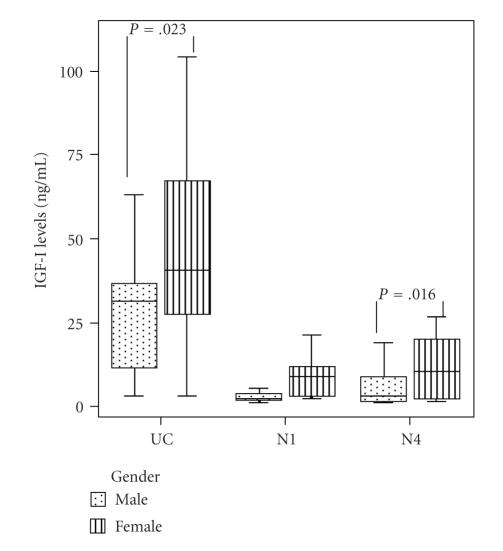
Box and whiskers plots of the concentration of IGF-I in fetal (UC), neonatal day-1 (N1), and day-4 (N4) serum samples from males and females. Each box represents the median concentration with the interquartile range (25th and 75th percentiles). The upper and lower whiskers represent the range.

Females presented elevated fetal and neonatal day-4 IGF-I levels (by 17.0 ng/mL and 7.5 ng/mL on average, resp.) compared to males (regression coefficient *b*: 17.0, SE: 7.1, *P* = .023, 95% CI: 2.5–31.5, and regression coefficient *b*: 7.5, SE: 2.9, *P* = .016, 95% CI: 1.5–13.5, resp.). Females also presented elevated day-1 IGF-I levels (by 3.8 ng/mL on average) compared to males (regression coefficient *b*: 3.8, SE: 1.9, *P* = .051, 95% CI: 0.02–7.5); however this difference was indicatively significant (Figure 3).

## 5. DISCUSSION

This study investigated concomitant changes of IGF-I and insulin at crucial perinatal time points in mother/infants pairs from IUGR and AGA pregnancies. It is known that IGF-I is a major hormonal determinant of fetal growth [15]; thus, IGF-I cord levels correlate with birth weight [16–19], a finding also recorded in both AGA and IUGR groups of our study granted that customized centiles represent adjusted birth weight [12, 13]. Moreover, reduced fetal IGF-I levels have been found to be predictive of intrauterine growth restriction [18, 20]. In this respect, we also demonstrated significantly lower fetal and neonatal day-1 IGF-I levels in the IUGR group.

A previous report stated that SGA newborns have lower IGF-I levels than AGA ones during the first week of life [11]. It has been suggested that reduced IGF-I levels in SGA infants may determine the rate of subsequent catchup growth [11]. In our study, significantly lower IGF-I levels were documented in IUGR day-1 neonates; however, this difference became statistically non significant on the 4th day postpartum, indicating a rise of serum IGF-I levels. Taken that IGF-I levels are an indicator of nutritional status [21] and low birth weight is known to lead to catchup growth in the neonate [22], we might speculate that catchup growth starts early after birth, provided that IUGR neonates are adequately fed, as was the case in our study (infants were breastfed ad libidum).

Up till now, many studies have failed to show a direct relationship between maternal serum IGF-I levels at term and birth weight in normal pregnancies [23, 24]. Holmes et al. reported an association between maternal IGF-I and birth weight in women with placental dysfunction, but not in healthy pregnant women [25]. Our data show a strong association between maternal circulating IGF-I levels and customized centiles in both normal and IUGR pregnancies. This is comparable with the finding of Boyne et al. who reported that maternal IGF-I levels increase with advancing gestation and correlate well with birth weight [4].

On the other hand, there is no prior evidence of transplacental transfer of maternal IGF-I, causing a direct growth-promoting/mitogenic effect on the fetus [4, 26]. The lack of correlation between maternal and fetal IGF-I, as well as the significantly higher IGF-I levels in maternal serum compared with cord blood in our study further support this concept. Hence, if maternal IGF-I does have an impact on fetal growth, the mechanism is probably mediated by increased nutrient supply, especially glucose, facilitating fetoplacental anabolism [4], as earlier suggested, based on the positive association between changes of IGF-I throughout gestation and placental size [24].

This study also shows a significant decrease of IGF-I on the first day postpartum in both IUGR and AGA groups. This finding could be attributed to the elimination of deciduas and placenta with birth, which represent two important sources of IGF-I during pregnancy [27].

It has been previously shown that IGF-I increases maternal insulin sensitivity, suppresses insulin production, and increases the transplacental transfer of glucose in an animal model [28]. In our study, a positive correlation between maternal IGF-I and insulin levels was demonstrated in the AGA group. The effects of circulating IGF-I on increasing insulin sensitivity are well recognized [29]. However, it has been postulated that IGF-I may have a further important role in maintaining *β*-cell mass and insulin secretory response to glucose [30].

On the other hand, in our IUGR group, maternal insulin levels were significantly elevated compared to fetal and neonatal days 1 and 4. This finding may be attributed to maternal pathologic conditions, associated with IUGR [31], as well as to the altered endocrine environment observed in these women during pregnancy [32]. These hormonal changes include increased maternal insulin levels, as reported in a low-protein animal model of intrauterine growth restriction [32].

Moreover, insulin has a central role in regulating fetal growth [9]. Body weight at birth is associated with the amount of functioning pancreatic tissue, with hyperinsulinemic babies being macrosomic and SGA babies having reduced *β*-cell mass [9]. A positive correlation between birth weight and cord insulin levels [16, 19] has been found, although some controversy exists [11, 33]. We report a strong correlation between fetal insulin levels and customized centiles.

In addition, insulin levels were lower on the first day of life in IUGR neonates compared to controls. This observation is in accordance with recent studies indicating that SGA newborns have significantly lower insulin levels and perhaps initially increased insulin sensitivity than AGA controls [34, 35]; this fact could possibly contribute to rapid early weight gain [36].

Finally, we observed, in both groups, a gender difference in the fetal and neonatal IGF-I concentrations, which were higher when the neonate was female, as has been described in older children [37, 38]. Results from measurements of IGF-I in serum of fetuses and preterm or term newborn infants have been more variable, as some groups find no difference in serum IGF-I concentrations between the male and female genders [39, 40]; others report higher IGF-I in girls than boys [41, 42]. However, this gender difference has not been, up till now, described in IUGR neonates. The reason for this gender difference is not clear, but it has been suggested that sex steroids could influence the secretion of IGF-I in utero [43]. Overall, our data indicate that a gender effect on IGF-I, which is produced by the fetoplacental unit, is operative in early life: the determinants and significance of this difference remain to be studied.

In conclusion, the positive correlations of maternal, fetal, and neonatal IGF-I levels, as well as fetal insulin levels with customized centiles underline the crucial role of both hormones in fetal growth. IUGR infants present lower, but gradually increasing, IGF-I levels, possibly predicting initiation of catchup growth. Insulin levels are lower in IUGR neonates on day 1 postpartum. Higher IGF-I levels in females may be attributed to influence of sex steroids in utero.

However, the exact mechanisms underlying the implications of IGF-I and insulin in the perinatal period of IUGR still need to be elucidated.

## Figures and Tables

**Table 1:** Demographic data of participating appropriate for gestational age (AGA) and intrauterine-growth-restricted (IUGR) mother/infant pairs.

	AGA (*n* = 20)		IUGR (*n* = 20)	
	Mean	Std. dev.	Mean	Std. dev.

Maternal age (years)	28	4	30.6	5.3
Parity
First	14 (70%)		11 (55%)	
Second	6 (30%)		7 (35%)	
Other	—		2 (10%)	

Gestational age (weeks)	38.20	0.90	38.10	0.70
Mode of delivery
Vaginal	12 (60%)		8 (40%)	
Cesarean section	8 (40%)		12 (60%)	

Birth weight (g)	3467	274.9	2342.5	228.57
Customized centile	75.6	7.56	1.5	1.5
Gender
Male	9 (45%)		11 (55%)	
Female	11 (55%)		9 (45%)	
